# Analysis of cellular and humoral immune responses against cytomegalovirus in patients with autoimmune Addison’s disease

**DOI:** 10.1186/s12967-016-0822-z

**Published:** 2016-03-09

**Authors:** Kine Edvardsen, Alexander Hellesen, Eystein S. Husebye, Eirik Bratland

**Affiliations:** Department of Clinical Science, University of Bergen, Laboratory Building, 8th floor, 5021 Bergen, Norway; Department of Medicine, Haukeland University Hospital, 5020 Bergen, Norway

**Keywords:** Addison’s disease, Antiviral immunity, Cytomegalovirus, Cytotoxic T lymphocytes

## Abstract

**Background:**

Autoimmune Addison’s disease (AAD) is caused by multiple genetic and environmental factors. Variants of genes encoding immunologically important proteins such as the HLA molecules are strongly associated with AAD, but any environmental risk factors have yet to be defined. We hypothesized that primary or reactivating infections with cytomegalovirus (CMV) could represent an environmental risk factor in AAD, and that CMV specific CD8^+^ T cell responses may be dysregulated, possibly leading to a suboptimal control of CMV. In particular, the objective was to assess the HLA-B8 restricted CD8^+^ T cell response to CMV since this HLA class I variant is a genetic risk factor for AAD.

**Methods:**

To examine the CD8^+^ T cell response in detail, we analyzed the HLA-A2 and HLA-B8 restricted responses in AAD patients and healthy controls seropositive for CMV antibodies using HLA multimer technology, IFN-γ ELISpot and a CD107a based degranulation assay.

**Results:**

No differences between patients and controls were found in functions or frequencies of CMV-specific T cells, regardless if the analyses were performed ex vivo or after in vitro stimulation and expansion. However, individual patients showed signs of reactivating CMV infection correlating with poor CD8^+^ T cell responses to the virus, and a concomitant upregulation of interferon regulated genes in peripheral blood cells. Several recently diagnosed AAD patients also showed serological signs of ongoing primary CMV infection.

**Conclusions:**

CMV infection does not appear to be a major environmental risk factor in AAD, but may represent a precipitating factor in individual patients.

**Electronic supplementary material:**

The online version of this article (doi:10.1186/s12967-016-0822-z) contains supplementary material, which is available to authorized users.

## Background

Autoimmune Addison’s disease (AAD) is an organ-specific autoimmune endocrinological disorder with uncertain pathogenesis caused by underlying environmental and genetic factors [1, 2]. AAD is characterized by highly specific immune responses against adrenocortical autoantigens, illustrated by autoantibodies against 21-hydroxylase (21OH), an enzyme involved in steroid hormone biosynthesis [3]. The pathological significance of these autoantibodies are uncertain however, it is assumed instead that 21OH-specific autoreactive T cells are the major players of the molecular pathogenesis of AAD. Both CD8^+^ and CD4^+^ 21OH-specific T cells have recently been described [4–6]. While the genetic background of AAD has been extensively characterized during the last decade, with numerous predisposing genetic variants discovered in genes encoding mostly immunologically relevant proteins [2, 7], almost nothing is known about the environmental risk factors. Viruses and other microbial pathogens are major environmental candidates as the hormone producing cells of the adrenal cortex are permissive to a wide range of infections, many of which can be clinically silent [8, 9]. Common viral agents known to infect the adrenal cortex and cause organ dysfunction include herpes simplex 1 and 2, Epstein-Barr virus, adenovirus, hepatitis B and cytomegalovirus (CMV) [9]. However, their possible involvement in the induction of autoimmune adrenalitis, and eventually overt AAD, is unclear.

The human cytomegalovirus, a member of the *herpesviridae* family, is a ubiquitous pathogen that persistently infects 60–90 % of the world’s population [10]. After a primary infection, which can be asymptomatic or cause a clinical picture resembling mononucleosis with fever, hepatitis, swollen lymph nodes and lymphopenia, the virus resides in latently infected monocytes and premonocytic cells with periodical reactivation driven by inflammation (e.g. increased levels of TNFα) or immunosuppression (e.g. HIV/AIDS or transplantation) [11, 12]. Primary or reactivating infections with CMV may cause severe disease in immunodeficient individuals and infants, and occasionally also in individuals with seemingly well-preserved immunity. During active CMV infection patients often suffer from immunological dysfunctions and autoimmune phenomena, such as autoantibodies [13–15]. In genetically predisposed individuals, primary CMV infections have been described as triggers of autoimmune disorders, such as vasculitides and scleroderma, which developed concomitantly with or immediately after active CMV infection in previously healthy, immunocompetent subjects [16–18]. Multiple case reports also describe primary, reactivating or persistent CMV infections as possible triggers of autoimmune endocrine diseases such as type 1 diabetes (T1D), Graves’ disease and AAD [19–22]. Importantly, CMV is known to infect and cause cytopathic damage to the adrenal cortex, and may directly cause adrenal insufficiency in infants and in immunodeficient individuals [23–26].

Since cellular immunity to specific viral agents have not been investigated in patients with AAD, the objective was to characterize the CD8^+^ T cell responses against specific HLA class I epitopes of CMV. In particular we wanted to look at HLA-B8 restricted anti-CMV responses, since HLA-B8 is the predominant HLA class I allele associated with AAD [27, 28]. This could also help to unravel possible gene-environment interactions jointly influencing the risk of developing AAD. We also found it interesting to investigate how a patient group with a dysregulated immune system, such as patients with AAD, would respond to a common virus such as CMV.

## Methods

### Patients and controls

The patient material [serum and peripheral blood mononuclear cells (PBMC)] used in the current study was accessed through the Norwegian registry and biobank for organ specific autoimmune diseases (ROAS). In total, 95 consecutively selected patients with confirmed AAD and known HLA-type (as described in [29]) were recruited. The control material was provided by the local blood bank and included 49 age- and gender-matched healthy controls. In addition, PBMC from 7 HLA-B8 positive healthy controls with confirmed positive CMV serostatus were purchased from Cellular Technology, Ltd (Shaker Heights, OH, USA) and included in the study. All patients and controls signed informed consent approved by the Health Region West ethics committee (149/96-47.96) and all experiments were conducted in accordance with the declaration of Helsinki. Heparinized blood samples were processed essentially as described previously [4]. In brief, plasma samples were isolated, aliquoted and kept frozen at −20 °C, while PBMC were isolated using Ficoll-Paque Plus (GE Healthcare, Little Chalfont, UK). The isolated PBMC were kept cryopreserved at −150 °C in 90 % human AB-serum (Lonza, Basel, Switzerland) and 10 % dimethylsulphoxide (DMSO) (Sigma-Aldrich, St Louis, MO, USA).

### CMV peptides and MHC dextramer reagents

HLA-A2 and -B8 restricted peptides, NLVPMVATV (from the pp65 antigen) and QIKVRVDMV (from the IE1 antigen), respectively, were purchased from Proimmune/thinkpeptides (Oxford, UK). MHC dextramers consisting of recombinant HLA-A2 and HLA-B8 and loaded with their respective cognate CMV peptides were purchased from Immudex (Copenhagen, Denmark), along with negative control MHC dextramers loaded with HIV gag-derived peptides SLYNTVATL (HLA-A2 restricted) and DIYKRWII (HLA-B8 restricted).

### HLA typing of whole blood

The correct HLA type (HLA-A2 or HLA-B8) of the healthy controls collected from the blood bank needed to be confirmed for inclusion in the downstream assays and this was performed using flow cytometry. 10 µL of FITC-conjugated anti-human HLA-A2 (clone BB7.2, Biolegend, San Diego, CA, USA) or PE-conjugated anti-human HLA-B8 antibodies (clone REA145, Miltenyi Biotech, Bergisch Gladbach, Germany) was added to 100 µL heparinized whole blood and incubated on ice for 30 min in the dark. Next, 2 mL of 1× lysisbuffer (BD Pharm lyse, BD Biosciences, San Jose, CA, USA) were added to each tube for a 10 min incubation at RT. The cells were then centrifuged for 5 min at 400×*g* and re-suspended in 2 mL phosphate-buffered-saline (PBS) with 1 % (w/v) bovine serum albumin (BSA, Sigma-Aldrich). The centrifugation step was repeated and the cells were re-suspended in 250 µL PBS with 1 % BSA and kept on ice in the dark until analyzed on an Accuri C6 flow cytometer. The gating procedure is described in Additional file 1: Figure S1.

### Determination of IgG and IgM antibodies to CMV in human serum

Sera or plasma from the patients and healthy controls selected for this study were examined for IgG or IgM antibodies to CMV using PLATELIA CMV immunoassay from BioRad (Hercules, CA, USA) according to the manufacturer’s protocol. Patients with positive IgG were included in the downstream assays.

### Cellular immune responses against CMV

Having determined the HLA type and CMV serostatus of our patients and controls, only those with antibodies to CMV and positive for either HLA-A2 (patients n = 6, controls n = 6) or –B8 (patients n = 9, controls n = 10) were selected for further investigation. Several different experiments were performed based on the amount of cells available. At day 0 PBMC stored at −150 °C were thawed, washed and re-suspended in serum free AIM V medium (Life-technologies, Paisley, UK) supplemented with 10 % human AB-Serum at 2 × 10^6^ cells/mL. 750 µL of the cell suspension was used for ex vivo CMV dextramer analysis (see below), 1200 µL was used in an ex vivo ELISpot assay and the remaining cells were seeded at 2 × 10^6^ cells/mL in 24-well culture plates for in vitro expansion. After having rested the cells for 24 h, at day 1 they were supplemented with 20 U/mL of IL-2 (Life-technologies) and stimulated with a final concentration of 1 µg/mL of B8 or A2 specific CMV peptide. At day 4, 6, 8 and 11, 1 mL of growth media was removed from the cells and 1 mL fresh media was added. At day 13 the cells were harvested and new dextramer and ELISpot assays were performed in addition to a degranulation assay. All assays are described in details below.

### CMV dextramer analysis

The 750 µL of 2 × 10^6^ cells/mL was divided between four tubes, three tubes with 167,000 cells and one tube with 1 × 10^6^ cells. 2 mL of wash buffer (PBS with 5 % FBS (Life-technologies)) were added to each tube and centrifuged at 300×*g* for 5 min. The cells were re-suspended in 50 µL wash buffer and 10 µL of CMV B8 or A2 specific dextramers (HLA-B*0801/QIKVRVDMV/PE and HLA-A*0201/NLPMVATV/PE) were added to the tube with 1 × 10^6^ cells. As a negative control HIV B8 or A2 specific dextramers (HLA-B*0801/DIYKRWII/PE and HLA-A*0201/SLYNTVATL/PE) were added to a second tube. The cells were incubated at RT in the dark for 10 min and then centrifuged as previously. The cells were re-suspended in 50 µL wash buffer and 5 µL of the following antibodies were added to three of the tubes (one tube was used as an unstained control), antihuman-CD8-APC (clone SK1, Biolegend, San Diego, CA, USA), antihuman-CD4-FITC (clone OKT4, Biolegend), antihuman-CD14-FITC (clone HCD14, Biolegend) and antihuman-CD19-FITC (clone HIB19, Biolegend), and incubated on ice in the dark for 20 min. 2 mL wash buffer was added and the cells centrifuged as previously, and the wash step repeated. The tubes with 167,000 cells were re-suspended in 200 µL wash buffer and the tube with 1 × 10^6^ cells were re-suspended in 400 µL wash buffer and kept on ice in the dark until analyzed on an Accuri C6 flow cytometer. The gating procedure is described in Additional file 2: Figure S2. This exact same procedure was performed on the cells that were harvested at day 13 after being stimulated with CMV peptide at day 1.

### ELISpot

The functional T cell response against B8 and A2 specific CMV peptides was tested in an IFN-γ enzyme linked immunospot (ELISpot^PRO^, Mabtech, Nacka Strand, Sweden) assay. The cells that were included at day 0 were rested for 24 h before 200 µL of the cell solution was added to six ELISpot wells, 4 × 10^5^ per well. One triplicate was stimulated directly with a final concentration of 1 µg/mL of a B8 or A2 specific CMV peptide, while the other triplicate was left untreated in medium only. After 24 h of incubation at 37 °C, the ELISpot assay was performed according to the manufacturer’s protocol. Spots were developed for 10 min, and after being washed and left to dry for 24 h, the wells were scanned by a Cellular Technology limited ELISpot counter and counted on the computer. The untreated cell spot count was subtracted from the stimulated cells. As positive controls confirming the viability of cells used in ex vivo ELISpot, activating anti-CD3 antibodies (clone CD3-2, mouse IgG2a, Mabtech) or phytohemagglutinin (PHA, Sigma-Aldrich) were used. These mitogenic stimuli generally resulted in spots too numerous to count. As negative controls, DMSO were added to control wells at the same concentration used for dissolving the CMV peptides. The same ELISpot procedure was performed on the in vitro expanded cells that were harvested at day 13, except the amount of cells added to each well was decreased to 5 × 10^4^. For cutoff limits we used the guidelines suggested by Chudley et al. [30] as follows: ELISpot data were first processed by expressing each well as spot forming units (SFU) per million PBMC, followed by subtracting the mean spot number of unstimulated cells from the stimulated cells. A positive response was acknowledged when the mean blank subtracted spot number of CMV stimulated cells exceeded 20 SFU per million PBMC for ex vivo ELISpot and 500 SFU per million PBMC for ELISpot post in vitro stimulation. The cutoffs always exceeded the mean of negative controls by at least two standard deviations. The actual presence of IFN-γ producing T cells in the ELISpot assays were confirmed by detection of IFN-γ by ELISA (see below) in supernatants of PBMCs (n = 3) stimulated with CMV peptides in parallel with ELISpot.

### Degranulation assay on stimulated PBMC

From the in vitro expanded cells harvested at day 13, 150 µL (300,000 cells) and 50 µL of fresh medium was added to four cell tubes. 1 µL of 200× Monensin (Biolegend) was added to each tube, while CMV peptide at a final concentration of 1 µg/mL was added to tube 1 and 2. 2 µL of antihuman-CD107a-FITC (clone H4A3, Biolegend) was added to tube 1 and 3, while 2 µL of FITC-conjugated isotype control (BD Biosciences) was added to tube 2 and 4. The tubes were incubated at 37 °C for 4 h. Next, 2 mL wash buffer was added to each tube, cells were centrifuged at 400×*g* for 5 min and re-suspended in 100 µL wash buffer. 5 µL of antihuman-CD8-APC was added to tube 1 and 3, and the cells were incubated on ice in the dark for 15 min. The wash step above was then repeated before the cells was re-suspended in 300 µL of wash buffer and kept on ice in the dark until analyzed on an Accuri C6 flow cytometer, essentially as described in [31].

### Interferon-γ ELISA

Serum levels of IFN-γ in a subset of AAD patients (n = 18) and controls (n = 11) were determined using a sandwich ELISA kit from Biolegend; Human IFN-γ ELISA MAX™ Deluxe, exactly as described by the manufacturer. This ELISA assay was also used for the defection of IFN-γ in cell-culture supernatants of CMV peptide stimulated cells, to confirm the presence of IFN-γ producing T cells in the ELISpot assays.

### Statistics

Statistical differences between patients and controls were calculated using non-parametric Mann–Whitney *U* test or Fisher’s exact test. For all statistical operations, *P* < 0.05 was considered significant. All tests were performed with GraphPad Prism v5.02.

## Results

### Antibodies to cytomegalovirus in AAD patients and healthy controls

**Fig. 1:**
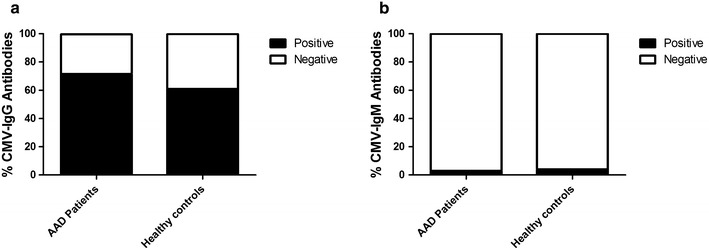
CMV IgG and IgM antibody levels. Platelia immunoassay was used to determine the frequencies of IgG (**a**), and IgM (**b**) antibodies to cytomegalovirus in serum/plasma samples from AAD patients (n = 95, mean age 40.1) and healthy controls (n = 49, mean age 42). The results are shown as percentage positive and negative subjects for each antibody. There were no significant differences in the frequencies of positive anti-CMV IgG or IgM between patients and controls as calculated by Fishers’ exact test

We measured the frequencies and levels of IgG and IgM CMV antibodies in 95 AAD patients (mean age 40.1, range 17–82) and 49 healthy controls (mean age 42.0, range 20–67). 71.6 % of the patients had CMV IgG antibodies (mean age 41.6), while for the healthy controls this number was lower at 61.2 % (mean age 42.8) (Fig. 1a). 3.2 % of the patients (mean age 34.7) had CMV IgM antibodies compared to 4.1 % for the healthy controls (mean age 28.5) (Fig. 1b). The differences in frequencies of CMV antibodies between patients and controls were not significantly different for either IgG or IgM (P = 0.26 and P = 1.0, respectively). The levels of CMV IgG are compared between patients and controls and shown in Additional file 3: Figure S3, although no significant differences were found (P = 0.53).

### Frequencies of CMV-specific CD8^+^ T cells

**Fig. 2:**
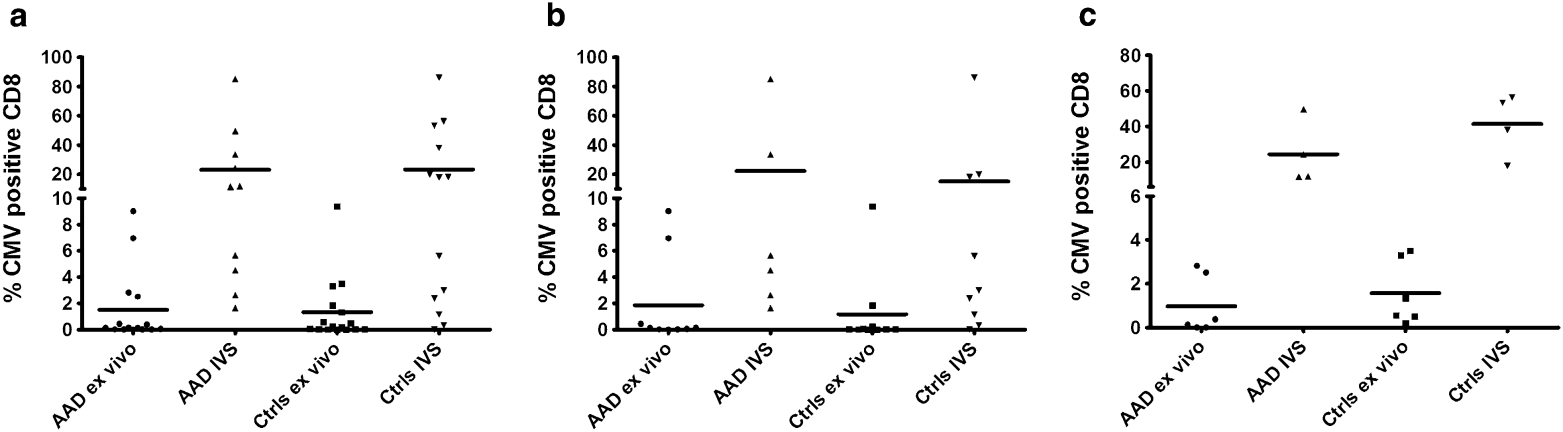
Ex vivo and in vitro stimulated (IVS) frequencies of CMV specific CD8^+^ T cells in AAD patients and controls. Immudex CMV dextramers were used to stain PBMC directly (ex vivo), or after the cells were stimulated with HLA-restricted CMV peptide (1 µg/mL) and expanded for 13 days (in vitro stimulated), for HLA-A2 or HLA-B8 CMV specific CD8^+^ T cells. All samples were analyzed on an Accuri C6 flow cytometer. Results are expressed as percentage of CD8^+^ T cells staining positive for CMV dextramers in **a** patients and controls. The same data are also shown grouped into HLA-B8 (**b**) and HLA-A2 (**c**) positive patients (**b** n = ex v. 9, IVS 6, **c** n = ex v. 6, IVS 4) and controls (**b** n = ex v. 10, IVS 9, **c** n = ex v. 6, IVS 4). Non-parametric Mann–Whitney *U* test was used to test for statistical differences between patients and controls, but none were found. The *bars* display the mean for the whole group

After having collected a cohort of patients and controls that had IgG antibodies to CMV and were either HLA-A2 (patients n = 6, controls n = 6) or HLA-B8 (patients n = 9, controls n = 10) positive, we wanted to investigate whether there were any differences in levels of CMV-specific CD8^+^ T cells in PBMC between the two groups. To this end, we used HLA-multimer technology to calculate the percentage of CMV-specific CD8^+^ T cells in patients and controls. No significant differences were found; either in PBMCs stained with HLA-dextramers ex vivo or in PBMCs stimulated and expanded in vitro (Fig. 2a). We also compared ex vivo and in vitro stimulated frequencies of CMV-specific CD8^+^ T cells with regard to HLA types, but no significant differences were found between patients and controls carrying either the AAD associated HLA-B8 subtype (Fig. 2b) or the common HLA-A2 subtype (Fig. 2c).

### Production of IFN-γ after stimulation with CMV peptides

**Fig. 3:**
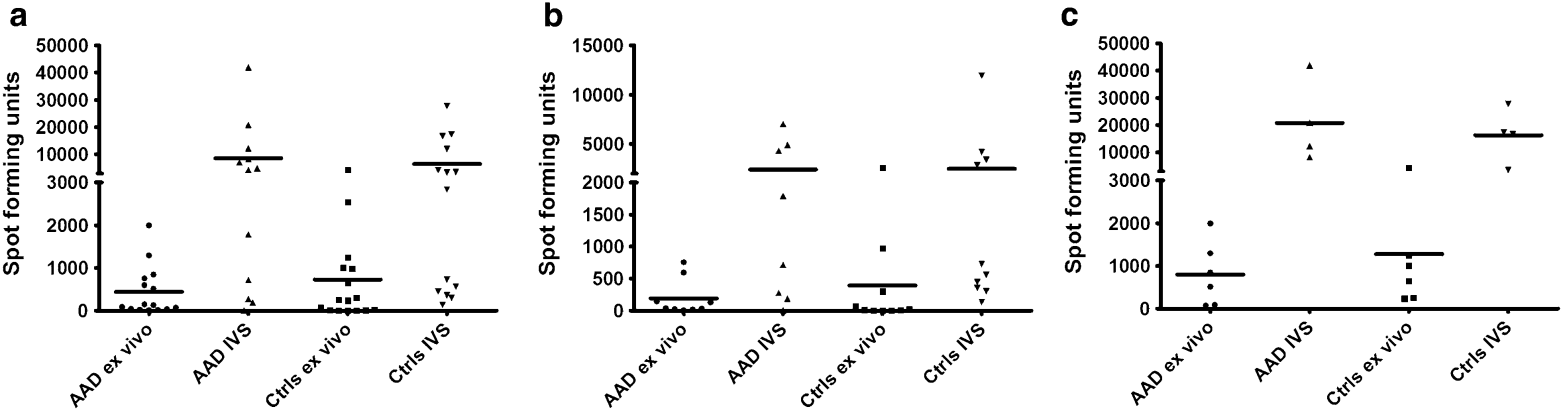
Ex vivo and in vitro stimulated (IVS) levels of CMV-specific IFN-γ producing T cells in AAD patients and controls. IFN-γ ELISpot was used to determine the amount of CMV-specific T cells in PBMC from HLA-A2 or HLA-B8 patients or healthy controls stimulated with HLA-restricted CMV peptide (1 µg/mL) for 24 h (ex vivo) or expanded for 13 days, and then then re-stimulated with CMV peptide for 24 h (in vitro stimulated). The results are displayed as means of triplicates of spot forming units (SFUs) per 1 × 10^6^ PBMCs (**a**). The same data are also shown grouped into HLA-B8 (**b**) and HLA-A2 (**c**) positive patients (**b** n = ex v. 9, IVS 8, **c** n = ex v. 6, IVS 4) and controls (**b** n = ex v. 10, IVS 10, **c** n = ex v. 6, IVS 4). Non-parametric Mann–Whitney *U* test was used to test for statistical differences between patients and controls, but none were found. The *bars* display the mean for the whole group

PBMCs from HLA-typed AAD patients and controls were stimulated with CMV peptides identical to the ones loaded onto the HLA dextramer reagents (restricted to either HLA-A2 or HLA-B8) and the resulting IFN-γ production was detected in an ELISpot assay. The assay’s capability to specifically detect IFN-γ was confirmed by ELISA analysis of IFN-γ in supernatants from PBMC cultures stimulated with CMV peptides in parallel to ex vivo ELISpot (n = 3). Mean (SD) levels were 453 (± 115) SFU/million PBMC for ELISpot and 1976 (± 110) pg/mL for ELISA, respectively. Similar to the HLA dextramer staining, ELISpot assays were performed both ex vivo and after in vitro stimulation and expansion. However, although mean IFN-γ SFU levels were slightly higher among the healthy controls, there were no significant differences between AAD patients and controls (Fig. 3a). Furthermore, no significant differences in the levels of IFN-γ production were detected in HLA-B8 positive (Fig. 3b) or HLA-A2 positive patients and controls (Fig. 3c).

### Cytolytic granule exocytosis in CMV-specific CD8^+^ T cells

**Fig. 4:**
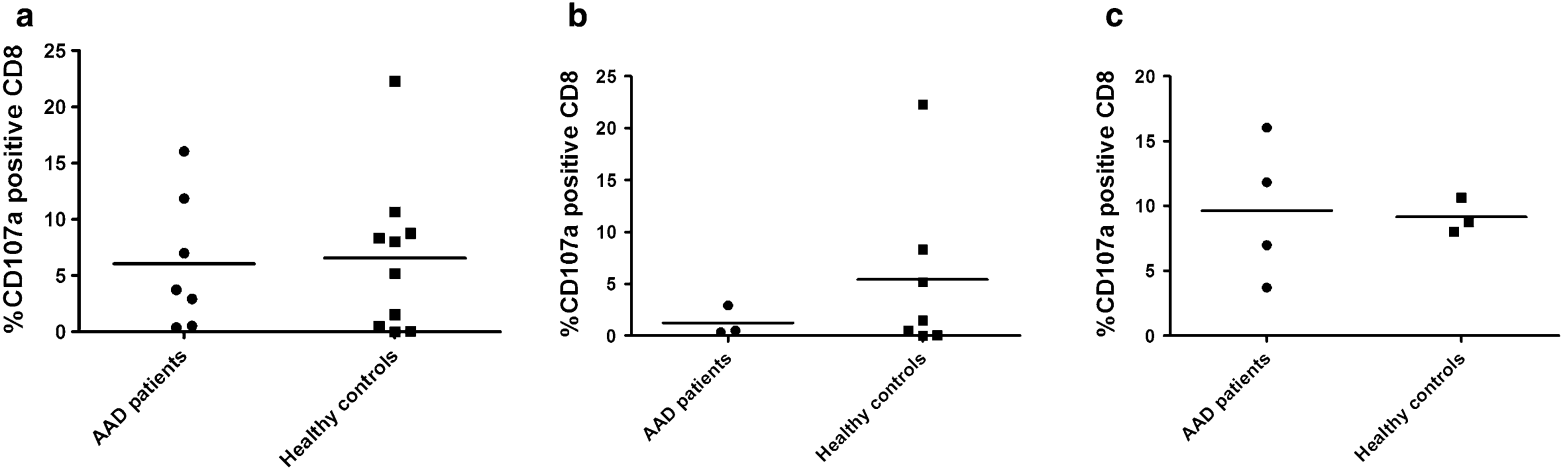
Levels of degranulating CMV-specific CD8^+^ T cells in AAD patient and control cells. PBMC from AAD patients and healthy controls were stimulated with HLA-specific CMV peptide (1 µg/mL) and expanded for 13 days, before being re-stimulated with CMV peptide and stained with anti-human CD107a for 4 h. The cells were also stained with anti-human CD8 before being analyzed on an Accuri C6 flow cytometer. Results are expressed as percentage of CD8^+^ T cells positive for CD107a in patients and controls (**a**). The same data are also shown grouped into HLA-B8 (**b**) and HLA-A2 (**c**) positive patients (**b** n = 3, **c** n = 4) and controls (**b** n = 7, **c** n = 3). Non-parametric Mann–Whitney *U* test was used to test for statistical differences between patients and controls, but none were found. The *bars* display the mean for the whole group

In a limited number of patients (n = 7) and controls (n = 10) the levels of degranulation after in vitro stimulation of PBMC were measured. To this end we measured the frequencies of CD107a positive CD8 cells after CMV peptide re-stimulation. Again, no significant differences could be found between AAD patients and healthy controls (Fig. 4a). Among the HLA-B8 individuals, the mean frequency of CD107a positive CD8^+^ T cells were higher among healthy controls than in AAD patients, but as the number of patients were low (n = 3) the difference was not significant (Fig. 4b). When comparing patients and controls with the HLA-A2 type, the frequencies of CD107a positive T cells in response to CMV stimulation were highly similar (Fig. 4c). Finally, we also compared the levels of mean fluorescence intensity (MFI) of CD107a staining of patient and control cells, but no statistical differences were found (P = 0.62, Additional file 4: Figure S4).

### AAD patients have decreased levels of circulating CD8^+^ T cells

**Fig. 5:**
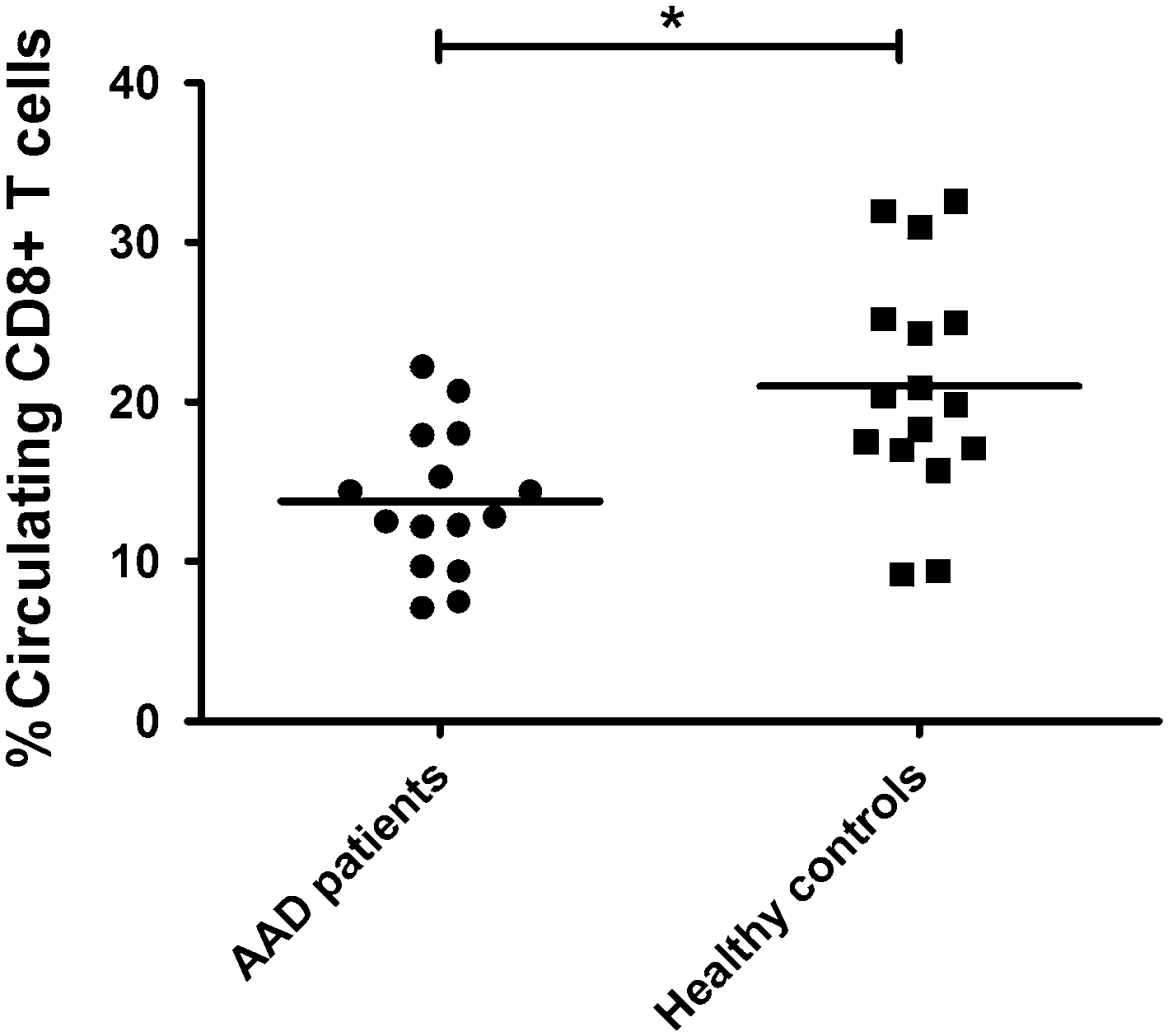
Levels of circulating CD8^+^ T cells are decreased in AAD patients. Flow cytometry was used to calculate the frequencies of circulating CD8^+^ T cells in PBMC from AAD patients (n = 15) and healthy controls (n = 16). Non-parametric Mann–Whitney *U* test was used to test for statistical differences between patients and controls, (*P < 0.05). The *bars* display the mean for the whole group

While investigating the ex vivo frequencies of HLA-dextramer positive CMV-specific T cells, we noticed that the AAD patients (n = 15) had decreased numbers of CD8^+^ T cells among their PBMCs compared to healthy controls (n = 16) (mean percent CD8^+^ T cells of total PBMC ± SD: 13.8 ± 4.5 vs 21.0 ± 7.1) (Fig. 5). This difference was highly significant (P = 0.005).

### Primary and reactivating CMV infections in individual AAD patients

**Fig. 6:**
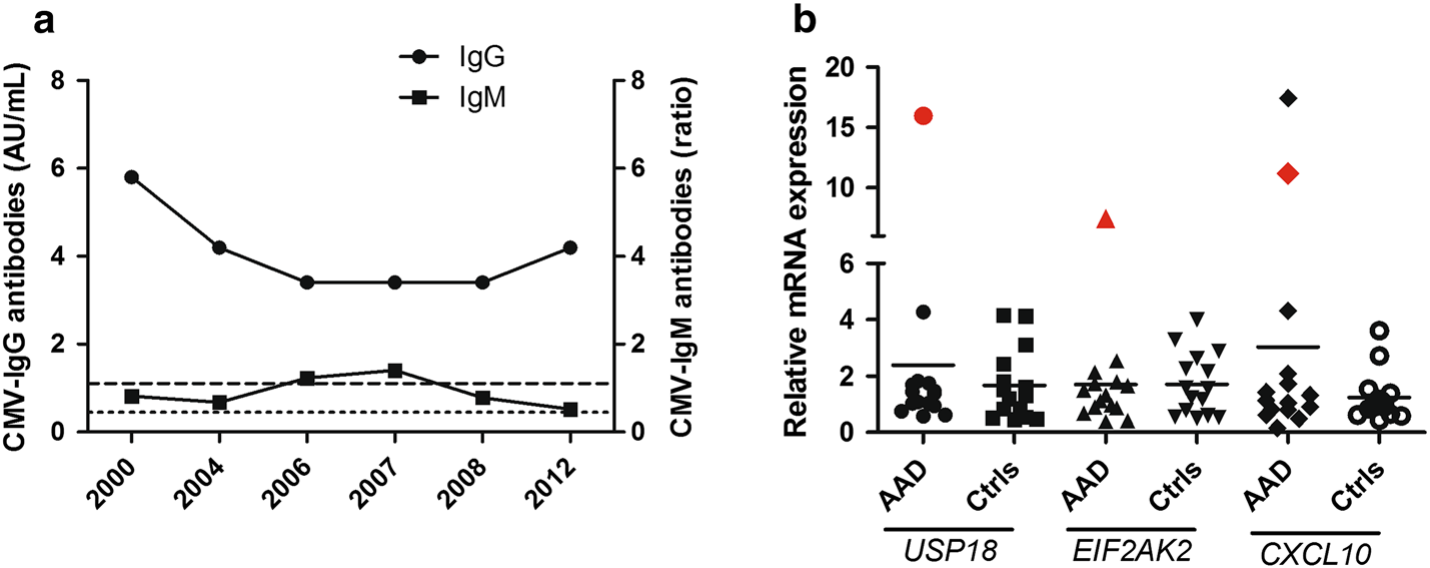
Reactivation of CMV infection and peripheral interferon signature in an AAD patient. Anti-CMV IgG [(*left y-axis* AU/mL)] and IgM [*right y-axis* (ratio between optical density and cut-off value)] levels are here shown for one individual patient over a 12 year time period (the patient was diagnosed with AAD in the year 2000) (**a**). IgG values are positive for the entire period (all values are above the *short stippled line*) while two IgM measurements are positive (values above the long stippled line) from 2006 to 2007. In **b** the relative mRNA expression in isolated CD4^+^ T cells of the three selected ISGs, *USP18*, *EIF2AK2* and CXCL10 previously reported in [32] is shown. The *red symbols* represent the same patient as in (**a**)

For 15 of the selected patients serum samples taken at various time points in the last 15 years were available, and longitudinal studies of anti-CMV antibodies and investigation of serological CMV status at the point of AAD diagnosis, were therefore possible. One patient, originally diagnosed with AAD in 2000 and with no other autoimmune co-morbidities, displayed a peculiar serological pattern. From 2000 through 2012 the anti-CMV IgG levels remained constantly positive. However, anti-CMV IgM antibodies were fluctuating (negative in 2000, positive in 2006–2007, negative in 2008, Fig. 6a), suggesting reactivating CMV infection from latency. This patient, who was HLA-B8 positive, had very few CMV-specific HLA-dextramer positive CD8^+^ T cells (less than 0.1 %). In a former study, we noticed that isolated CD4^+^ T cells from the patient in question displayed strongly elevated mRNA levels of the IFN-regulated genes Ubiquitin Specific Peptidase 18 (*USP18*), Eukaryotic translation Initiation Factor 2-Alpha Kinase 2 (*EIF2AK2*) and C-X-C motif chemokine 10 (*CXCL10*) compared to other AAD patients and healthy controls [32], indicating an IFN signature in her peripheral blood cells (illustrated here in Fig. 6b). Furthermore, the daughter of this patient (also HLA-B8 positive) developed AAD in 2012 and, despite of being anti-CMV IgG positive, had no detectable CMV-specific HLA dextramer positive cells. As expected, both mother and daughter had very low numbers of IFN-γ producing cells after CMV peptide stimulation (38 and 30 SFU/million PBMC, respectively). After in vitro stimulation, the CMV specific cells of the mother could be expanded and reached a very high percentage (85.1 %) of HLA dextramer positive cells. However, the frequency of IFN-γ producing cells were still relatively low at 5927 SFU/million cells, given the high frequency of dextramer positive cells. The CMV specific cells of the daughter could not be expanded after in vitro stimulation. The cells of both the mother and daughter responded vigorously to anti-CD3 stimulation, however, excluding poor viability as an explanation for the observed results.

Two patients were positive for anti-CMV IgM at the time of AAD diagnosis (one of them also positive for IgG), suggesting primary CMV infections as a precipitating factor for adrenal insufficiency in these patients. One additional patient became anti-CMV IgM positive 2 months after AAD diagnosis, with subsequent seroconversion to positive anti-CMV IgG and negative anti-CMV IgM 1 year later. These results are summarized in Table 1.

**Table 1:** Summary of patients positive for IgM close to AAD diagnosis

AAD patient number	Age at diagnosis	CMV serostatus at diagnosis		IFN-γ levels (IgM) (pg/mL)
		IgG	IgM	
1	25	Positive	Positive	116.4
2	30	Negative Positive post 6 months	Negative Positive post 2 months	282.4
3	32	Negative	Positive	4.0

**Fig. 7:**
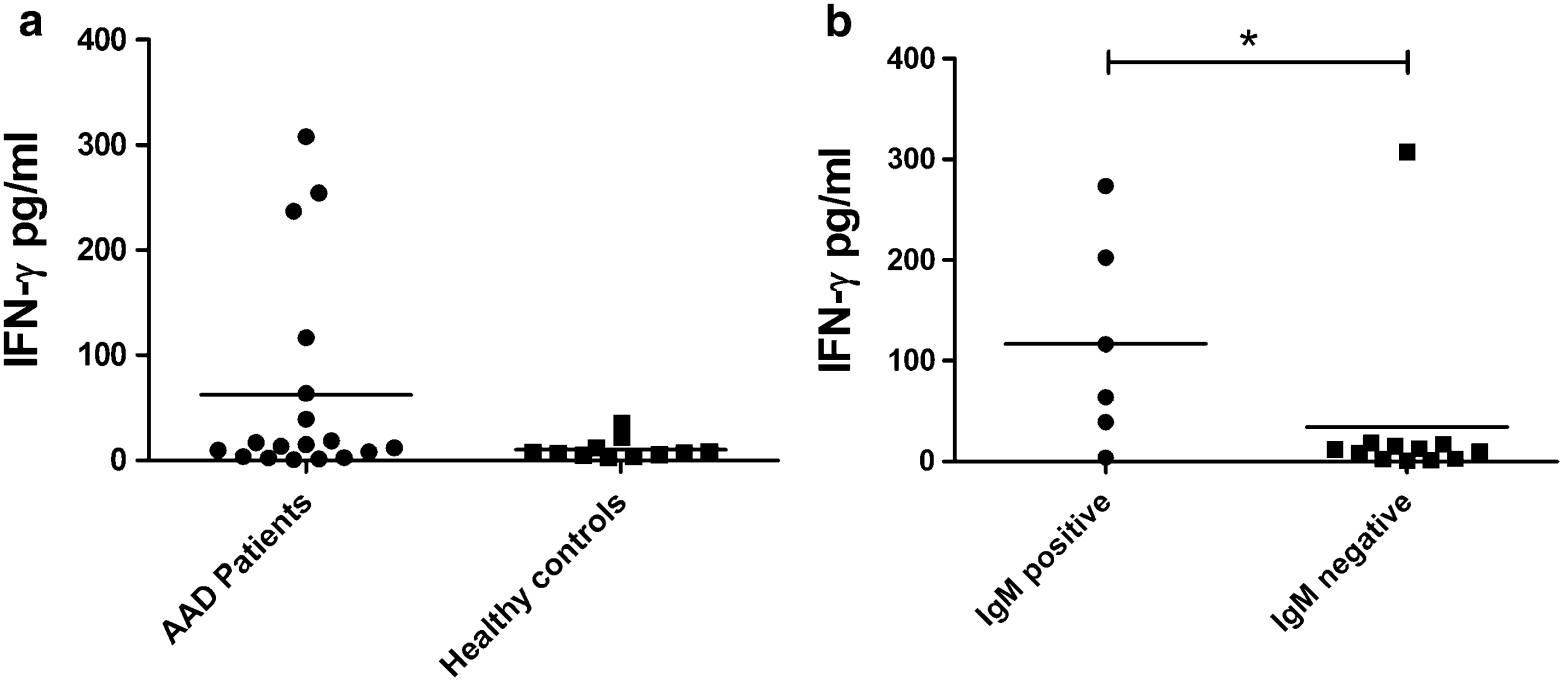
Increased serum IFN-γ levels in patients positive for anti-CMV IgM antibodies. Serum IFN-γ levels were determined in 18 AAD patients and 11 healthy controls using sandwich ELISA (**a**). In **b** serum IFN-γ levels were compared between those patients positive for anti-CMV IgM antibodies (n = 6), with those that were negative (n = 12). Non-parametric Mann–Whitney *U* test was used to test for statistical differences (*P < 0.05). The* bars *display the mean for the whole group

Serum IFN-γ levels were determined in a subset of patients (n = 18) and controls (n = 11), including all samples positive for anti-CMV IgM antibodies. No statistical differences were found between patients and controls (Fig. 7a), although individual patients had considerable elevated IFN-γ levels. However, when comparing the serum IFN-γ levels in patients with IgM antibodies to the patients without IgM antibodies, a significant difference was found (P = 0.035, Fig. 7b). Notably, the patient described in Fig. 6 with signs of CMV reactivation and an IFN signature in her peripheral blood, were among the patients with highest serum levels of IFN-γ (254 pg/mL).

## Discussion

It has been suggested that virus infections may provoke AAD in genetically susceptible individuals [33, 34]. Direct destruction of adrenocortical cells by virus infection, immune mediated destruction by cross-reacting T cells or non-specific enhancement of antigen presentation has been suggested to be the underlying pathogenic mechanisms [35]. CMV infection is one of the most common viral infections throughout the world and has been associated with a number of autoimmune diseases, including systemic lupus erythematosus (SLE), T1D, vasculitis and Sjögren’s syndrome [16, 21, 36, 37]. Even though a healthy immune system controls CMV infection, the virus can never be eliminated by immune effector mechanisms or antiviral drugs. Thus, CMV establishes persistent life-long infections and may reactivate periodically, possibly representing an environmental risk factor for autoimmune diseases. Primary, latent and reactivating CMV infections are controlled immunologically by a number of molecular and cellular effector systems, including CD4^+^ and CD8^+^ T cells, NK cells, type I and II IFNs [38]. Antibodies targeting CMV virion surface antigens are essential in limiting dissemination of recurrent virus. Individuals infected with CMV are left with permanent imprints affecting their cellular immune system, in particular among the CD8^+^ T cells, where up to 20 % of circulating cells may be specific for CMV epitopes [39, 40]. For the current study, we therefore chose to focus on the CD8^+^ T cell compartment in order to assess CMV-specific immunity in AAD patients.

Individuals that have undergone primary CMV infection usually remain positive for CMV-specific IgG antibodies throughout life. The transient presence of CMV-specific IgM antibody has long been used as a diagnostic marker for primary CMV infection, but IgM can also be present during viral reactivation or reinfection [41]. In the current study, we initially determined and compared the frequencies of anti-CMV IgG and IgM antibodies in a relatively large cohort of AAD patients and age-matched healthy controls. Although frequencies of anti-CMV IgG antibodies were slightly higher among patients compared to controls, the difference was not statistically significant. Similarly, when comparing the levels of anti-CMV antibodies, no differences were found between patients and controls. Still, in related studies of other autoimmune disorders and their association to viral infections (e.g. multiple sclerosis (MS) and Epstein–Barr virus, or SLE and CMV), a higher number of sera (>100) were needed to establish a statistical significant relationship between seropositivity and autoimmune disease [42, 43]. Therefore, a follow-up study with a substantially increased number of AAD patient and control sera could still reveal possible associations between AAD and CMV.

After having identified patients seropositive for anti-CMV IgG, we went on to assess whether any defects in their cellular immunity against CMV could be detected. Assuming that a suboptimal control of CMV in AAD patients may be reflected in a decreased number of CMV-specific CTLs, we have analyzed the frequencies of HLA-B8 and –A2 restricted CTLs in patients and healthy controls using HLA-dextramers and IFN-γ ELISpot assays. T cell responses were investigated both ex vivo and after in vitro stimulation in order to include both effector and central memory T cells. The ex vivo analysis clearly demonstrates that the frequency of CMV-specific CD8^+^ T cells in AAD patients are within the range normally observed in otherwise healthy virus carriers. Similarly, no differences could be detected in the frequencies of CMV-specific CD8^+^ T cells, or in their capacity to degranulate upon antigen stimulation after in vitro stimulation and expansion. Overall, our data therefore do not support any general defects in AAD patients’ effector of memory CTL response to CMV. Still, this is an important finding since it indicates that the treatment regime for AAD, oral glucocorticoid supplementation therapy, does not have a significant functional effect on the in vivo expansion or the IFN-γ production profile of CMV-specific T cells.

Since HLA-B8 is among the strongest predisposing genetic factors associated with AAD and a number of related autoimmune diseases [28, 44, 45], we were particularly interested in T cell responses restricted by this allele. A suspected consequence of such genetic associations to autoimmune diseases is perhaps the propensity of the allele in question to present certain self-peptides [46]. Indeed, a certain peptide of 21-hydroxylase (21OH), the major antigen in AAD, has been identified as being presented by HLA-B8 to autoreactive CD8^+^ T cells from AAD patients [5, 6]. However, these alleles may also be involved in gene-environment interactions, presenting peptides from viral or infectious agents in an aberrant or deficient manner. We found no differences between HLA-B8 positive AAD patients and controls in frequencies of ex vivo or in vitro expanded CMV-specific T cells, or in their abilities to produce IFN-γ or degranulate. A similar picture emerged when comparing AAD patients and healthy controls carrying the common MHC class I variant HLA-A2. We did notice that HLA-A2 restricted responses in general tended to be stronger than HLA-B8 restricted responses. This pattern was evident both ex vivo and in in vitro expanded cells for both AAD patients and controls. In fact, when all cellular samples were grouped according to HLA type regardless of disease status, HLA-A2 restricted responses were significantly stronger than HLA-B8 restricted responses (data not shown). This must be interpreted with caution since there are several immunodominant CMV-specific epitopes that are presented by both HLA-A2 and HLA-B8 [47, 48], and in the current study we only had the opportunity to assess one epitope per HLA type. However, a similar scenario has been described for HLA-B8 restricted T cells from healthy donors and their relative insufficiency to lyse EBV infected B cells, compared to T cells restricted to other HLA types [49]. HLA-B8 is also part of the ancestral MHC haplotype 8.1 (consisting of HLA-A1, -B8, -C7, -DR3 and -DQ2), which has been described to affect early stages of lymphocyte activation and modulate cytokine production [45]. In particular, IFN-γ production is reduced in carriers of the 8.1 haplotype [50].

When analyzing the HLA-dextramer data, we noticed that the AAD patients had significantly fewer circulating CD8^+^ T cells among their PBMCs than healthy controls. To our knowledge this has never been reported for AAD previously, but has been recognized for many years as a general feature of several autoimmune diseases (reviewed in [51]). The deficiency in CD8^+^ T cells is probably genetically determined since it also occurs in healthy family members of patients with autoimmune diseases [52]. The significance of this general phenomenon is currently not known, but redistribution and sequestration of the CD8^+^ T cells to the target organ is one of the suggested possibilities [53]. Another hypothesis is that the deficiency of CD8^+^ T cells impairs the control of EBV infection, resulting in clonal expansion of autoreactive EBV infected B cells in the target organ followed by infiltration of autoreactive T cells and extensive tissue damage [51].

Even though AAD patients in general had normal cellular and humoral immune responses to CMV, some deviations were found in individual patients. Three patients were anti-CMV IgM positive close to the point of AAD diagnosis. Two of them were also IgG negative suggesting that CMV primary infection were involved as a triggering or precipitating factor. This complements a recent case report describing a young female developing AAD shortly after undergoing verified CMV infectious mononucleosis [19]. Another patient initially only positive for IgG became IgM positive for a period of 2 years, suggesting reactivating or secondary CMV infection. Strikingly, this patient also had very few circulating CMV-specific T cells and consequently very poor IFN-γ production in response to CMV peptides. The patient also had an IFN signature in her peripheral blood cells, and very high serum levels of IFN-γ. We speculate that the latter may be a consequence of persistent CMV infection since the CMV virion associated factors recognized by host cells as foreign pathogen-associated molecular patterns (PAMPs) activate IRF3, NFκB and an overall transcriptional profile similar to that observed following IFN treatment [54]. These speculations were also supported by the fact that this patient, and also most of the other AAD patients with IgM antibodies against CMV, had very high serum levels of IFN-γ. Such high systemic IFN-γ levels have previously been described to reflect primary CMV infection [55]. Finally, the daughter of the patient mentioned above also developed AAD, and displayed the same anti-CMV cellular patterns: low levels of CMV-specific HLA-dextramer positive cells, and very low IFN-γ production after stimulation with CMV peptide, in spite of being anti-CMV IgG positive. All in all, the findings in this mother and daughter suggest an inheritable immunological phenotype with susceptibility of developing AAD and decreased cellular immunity against CMV. Still, it must also be mentioned that lacking CD8^+^ T cell responses to CMV peptides were also observed among the controls, even in the presence of positive anti-CMV IgG, as other have reported previously [56].

Both CD4^+^ and CD8^+^ T cells, NK cells and antibodies against surface antigens play a crucial role in the immune defense against CMV, preventing the development of CMV disease in the immunocompetent host [38]. Any patient-specific deficiencies in controlling CMV infection may therefore be inherent to any of these cellular subsets, and not just to CD8^+^ T cells that were the main focus of the present study. Furthermore, because the experimental approaches used to investigate CTLs in our current investigation do not include subset-specific analysis of CD8^+^ T cells, we cannot exclude that such differences might have escaped detection. In patients with MS, a deficiency in CD8^+^ effector memory cells has been described as a feature that may underlie poor control of EBV infection [57]. A more in depth analysis of the cytolytic capabilities of CMV specific CD8^+^ T cells is also warranted, since the degranulation assay we have performed in the current study only included a limited number of patients and controls and lacked a proper positive control such as staphylococcal enterotoxin B (SEB).

In order to fully investigate CMV immunity in AAD patients, CD4^+^ T cell immunity should also be assessed in future studies. The importance of such studies is underlined by the fact that the strongest genetic factors associated with the risk of developing AAD are certain HLA class II alleles [28, 29]. In particular, it would be of high interest to analyze the presence of CD4^+^CD28^−^ T cells, which have been implicated in both autoimmune diseases like rheumatoid arthritis, MS and Graves’ disease [58–60], but also specifically in relation to CMV infection [61].

## Conclusion

We could not find evidence that CMV infection is a major player in the pathogenesis of AAD. In general, both humoral and CD8^+^ T cell mediated immunity to CMV are normal in patients with AAD. However, as AAD is a multifactorial disorder we find it likely that common infections with persistent viral agents such as CMV may contribute as a triggering or precipitating factor in individual patients. The genetic susceptibility to develop autoimmune diseases such as AAD may also go hand in hand with slightly less robust immunity to common pathogens such as CMV and other herpesviruses.

## Additional files

10.1186/s12967-016-0822-z Gating strategy for flow cytometric analysis of HLA-stained whole blood from healthy blood donors. Dot plots show the gating strategy used to determine the HLA type of the blood donors. P1: Gating on lymphocyte population R1: HLA-A2 (FITC) positive cells in P1. R2: HLA-B8 (APC) positive cells in P1.
10.1186/s12967-016-0822-z Gating strategy for flow cytometric analysis of dextramer-stained PBMC from AAD patients and healthy controls. Dot plots show the gating strategy used to determine the levels of dextramer positive CD8^+^ T cells. P1: singlet selection to avoid cellular doublets. R1: Positive gating of CD8^+^ population (using APC-conjugated antibodies) in P1. Exclusion of CD4^+^ T cells, CD14^+^ monocytes and CD19^+^ B cells (using FITC-conjugated antibodies), to avoid unspecific dextramer binding. P2: Positive gating on R1 population, excluding dead cells. R2: Positive gating on P2 population, selecting CMV-dextramer (PE-conjugated) positive CD8^+^ T cells.
10.1186/s12967-016-0822-z CMV IgG antibody levels. The anti-CMV IgG antibody levels shown as arbitrary units per milliliter (AU/mL) from AAD patients (n = 65) and healthy controls (n = 29) positive for anti-CMV IgG antibodies. The antibody levels at the y-axis are shown logarithmically. Non-parametric Mann–Whitney *U* test was used to test for statistical differences between patients and controls, but none were found. The *bars* display the mean for the whole group.
10.1186/s12967-016-0822-z Mean fluorescent intensity (MFI) of degranulating CMV-specific CD8^+^ T cells in AAD patients and controls. The specific mean fluorescent intensity (ΔMFI) of CMV stimulated CD107a positive cells (after subtracting the MFI of unstimulated cells) was compared between patients (n = 7) and controls (n = 10). Unpaired t test was used to test for statistical differences between patients and controls, but none were found. The *bars* display the mean for the whole group.
