# Supplementary figures and images for: Analysis of cellular and humoral immune responses against cytomegalovirus in patients with autoimmune Addison’s disease
Source: J Transl Med. 2016 Mar 9;14:68. doi: 10.1186/s12967-016-0822-z (PMC4784442; doi:10.1186/s12967-016-0822-z)

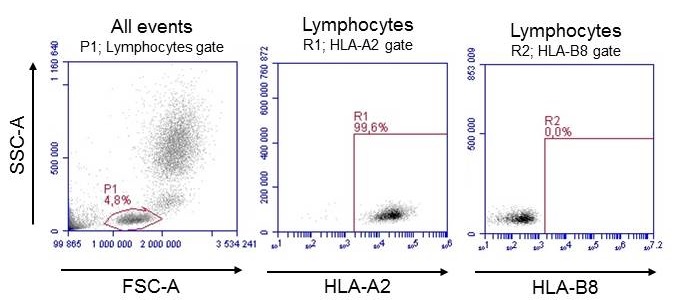

Supplement: Supplementary file 1 — 10.1186/s12967-016-0822-z Gating strategy for flow cytometric analysis of HLA-stained whole blood from healthy blood donors. Dot plots show the gating strategy used to determine the HLA type of the blood donors. P1: Gating on lymphocyte population R1: HLA-A2 (FITC) positive cells in P1. R2: HLA-B8 (APC) positive cells in P1. [file 12967_2016_822_MOESM1_ESM.jpg]

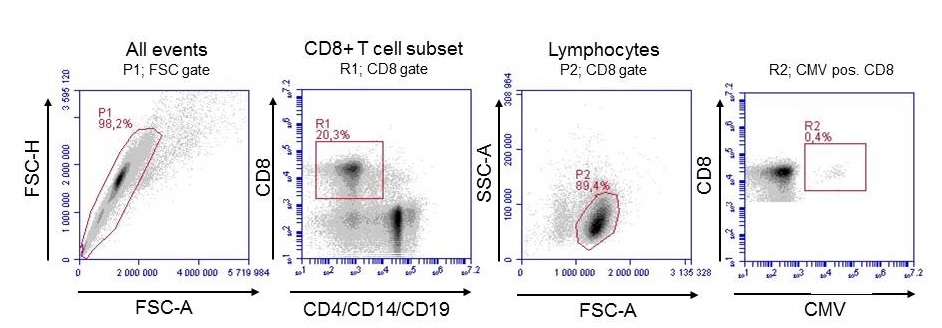

Supplement: Supplementary file 2 — 10.1186/s12967-016-0822-z Gating strategy for flow cytometric analysis of dextramer-stained PBMC from AAD patients and healthy controls. Dot plots show the gating strategy used to determine the levels of dextramer positive CD8+ T cells. P1: singlet selection to avoid cellular doublets. R1: Positive gating of CD8+ population (using APC-conjugated antibodies) in P1. Exclusion of CD4+ T cells, CD14+ monocytes and CD19+ B cells (using FITC-conjugated antibodies), to avoid unspecific dextramer binding. P2: Positive gating on R1 population, excluding dead cells. R2: Positive gating on P2 population, selecting CMV-dextramer (PE-conjugated) positive CD8+ T cells. [file 12967_2016_822_MOESM2_ESM.jpg]

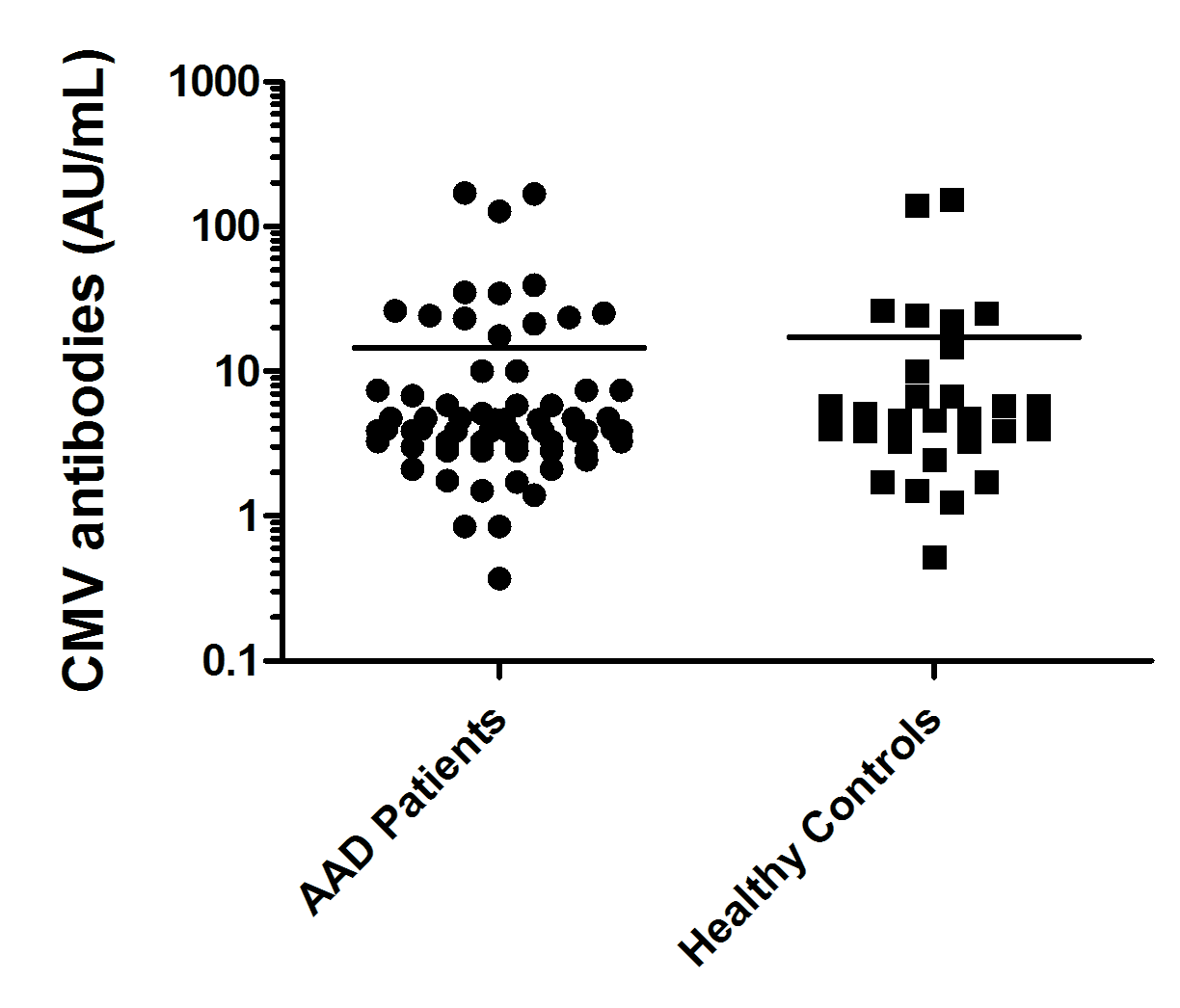

Supplement: Supplementary file 3 — 10.1186/s12967-016-0822-z CMV IgG antibody levels. The anti-CMV IgG antibody levels shown as arbitrary units per milliliter (AU/mL) from AAD patients (n = 65) and healthy controls (n = 29) positive for anti-CMV IgG antibodies. The antibody levels at the y-axis are shown logarithmically. Non-parametric Mann–Whitney U test was used to test for statistical differences between patients and controls, but none were found. The bars display the mean for the whole group. [file 12967_2016_822_MOESM3_ESM.tif]

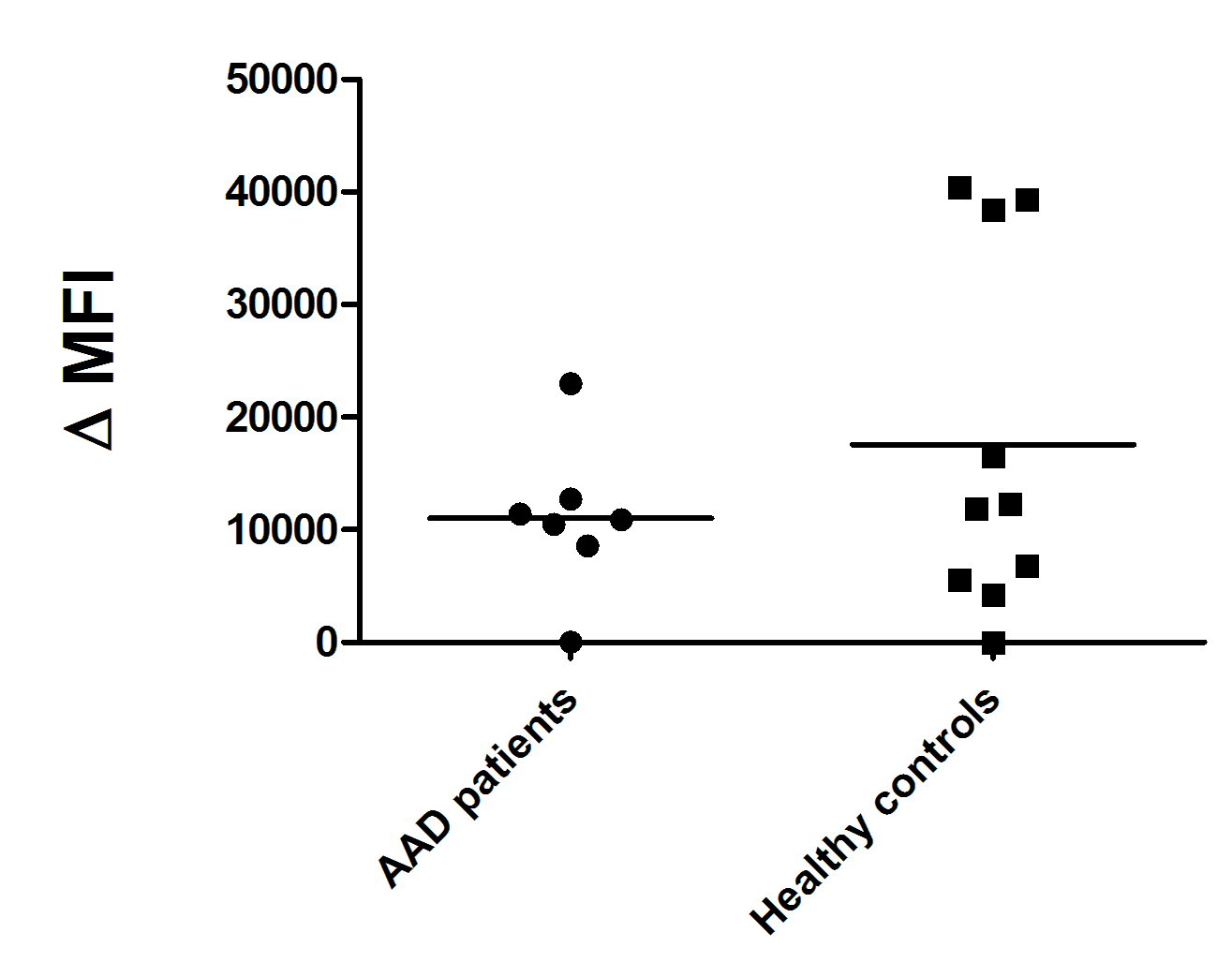

Supplement: Supplementary file 4 — 10.1186/s12967-016-0822-z Mean fluorescent intensity (MFI) of degranulating CMV-specific CD8+ T cells in AAD patients and controls. The specific mean fluorescent intensity (ΔMFI) of CMV stimulated CD107a positive cells (after subtracting the MFI of unstimulated cells) was compared between patients (n = 7) and controls (n = 10). Unpaired t test was used to test for statistical differences between patients and controls, but none were found. The bars display the mean for the whole group. [file 12967_2016_822_MOESM4_ESM.tif]
